# Sacro pit: a rare clinical image

**DOI:** 10.11604/pamj.2023.45.168.40974

**Published:** 2023-08-18

**Authors:** Dharti Meshram, Pooja Kasturkar

**Affiliations:** 1 Department of Mental Health Nursing, Smt. Radhikabai Meghe Memorial School of Nursing (FNTCN), Datta Meghe Institute of Medical Sciences (Deemed to be University), Sawangi, Wardha, Maharashtra, India,; 2 Department of Mental Health Nursing, Smt. Radhikabai Meghe Memorial College of Nursing, Datta Meghe Institute of Higher Education and Research (Deemed to be University), Sawangi, Wardha, Maharashtra, India

**Keywords:** Sacral dimple, spinal dysraphism, infant, lumbosacral region, magnetic resonance imaging

## Image in medicine

A pit or indentation in the coating of the lower back is known as a sacral dimple. Small and shallow sacral dimples are the norm. 1.8%-7.2% of newborns have a sacral dimple. If a dimple is evident, less than 0.5 cm in size, and has one midline lesion, it has allegedly been described as a typical benign lesion. A sacral dimple is not known to have any known causes. Treatment for spinal issues depends on what caused them in the first place. Sacral dimples are often not problematic. A sacral dimple may occasionally indicate an underlying spinal condition. These include tethered spinal cord and spina bifida. Despite some claims to the contrary and scant scientific investigation of dimples. Therefore, it is unknown which genes may contribute to dimples. Most sacral dimples are harmless and do not require any care. We here reported 6 months of infant female who came to paediatric Outpatient Department (OPD) with mother and known case of a congenital sacro pit in the skin on the lower back buttocks. Where is no any history of irritation, discharge, pain, passage of fluids or fever. Patient came for further management. Physical examination done and found that sacro pit. The patient is conscious. Call was noted to neurosurgeon and paediatric surgeon. Ultrasonographic (USG) and Magnetic Resonance Imaging (MRI) done noted that 3mm sacro pit. Treatment given in hospital Syp. Zincovit Syp. Vit D3. Patient general condition is good. No any other management given to child regarding sacro pit. Patient was discharged. Doctor Advise to patient family do regular follow-up if any issues occur regarding this. Patient is healthy.

**Figure 1:**
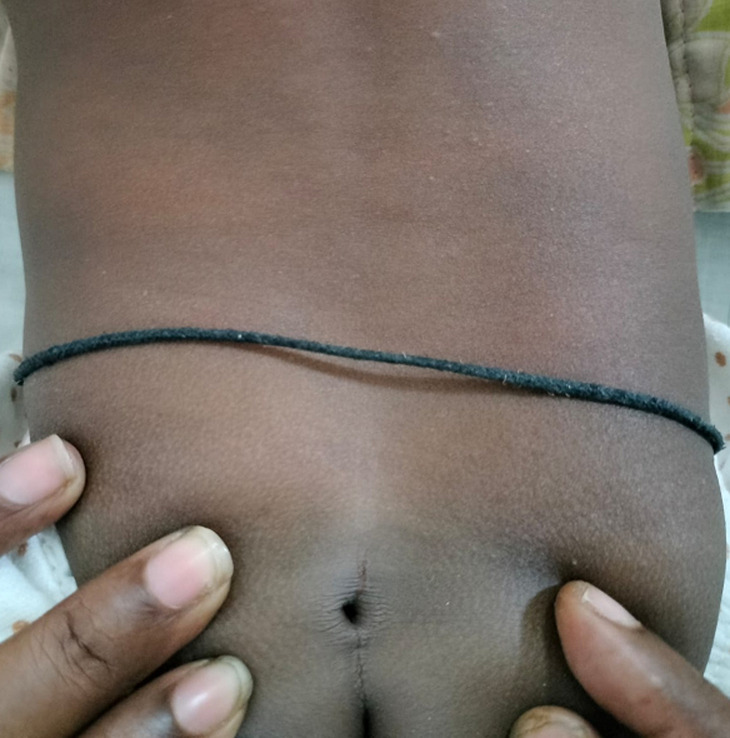
pit on sacral region

